# Prognostic Impact of Tumor-Associated Macrophages on Survival Is Checkpoint Dependent in Classical Hodgkin Lymphoma

**DOI:** 10.3390/cancers12040877

**Published:** 2020-04-04

**Authors:** Kristiina Karihtala, Suvi-Katri Leivonen, Oscar Brück, Marja-Liisa Karjalainen-Lindsberg, Satu Mustjoki, Teijo Pellinen, Sirpa Leppä

**Affiliations:** 1 Applied Tumor Genomics Research Program, Faculty of Medicine, University of Helsinki, 00014 Helsinki, Finland; kristiina.karihtala@hus.fi (K.K.); suvi-katri.leivonen@helsinki.fi (S.-K.L.); 2 Department of Oncology, Helsinki University Hospital Comprehensive Cancer Center, 00029 Helsinki, Finland; 3 Translational Immunology Research Program and Department of Clinical Chemistry and Hematology, University of Helsinki, 00014 Helsinki, Finland; oscar.bruck@helsinki.fi (O.B.); satu.mustjoki@helsinki.fi (S.M.); 4 Hematology Research Unit Helsinki, Helsinki University Hospital Comprehensive Cancer Center, 00029 Helsinki, Finland; 5 Department of Pathology, Helsinki University Hospital, 00029 Helsinki, Finland; marja-liisa.karjalainen-lindsberg@hus.fi; 6 Institute for Molecular Medicine Finland (FIMM), 00014 Helsinki, Finland; teijo.pellinen@helsinki.fi

**Keywords:** classical hodgkin lymphoma, tumor-associated macrophages, tumor microenvironment, checkpoint molecules, multiplex immunohistochemistry, survival

## Abstract

Tumor microenvironment and immune escape affect pathogenesis and survival in classical Hodgkin lymphoma (cHL). While tumor-associated macrophage (TAM) content has been associated with poor outcomes, macrophage-derived determinants with clinical impact have remained undefined. Here, we have used multiplex immunohistochemistry and digital image analysis to characterize TAM immunophenotypes with regard to expression of checkpoint molecules programmed cell death ligand 1 (PD-L1) and indoleamine 2,3-dioxygenase 1 (IDO-1) from the diagnostic tumor tissue samples of 130 cHL patients, and correlated the findings with clinical characteristics and survival. We show that a large proportion of TAMs express PD-L1 (CD68^+^, median 32%; M2 type CD163^+^, median 22%), whereas the proportion of TAMs expressing IDO-1 is lower (CD68^+^, median 5.5%; CD163^+^, median 1.4%). A high proportion of PD-L1 and IDO-1 expressing TAMs from all TAMs (CD68^+^), or from CD163^+^ TAMs, is associated with inferior outcome. In multivariate analysis with age and stage, high proportions of PD-L1^+^ and IDO-1^+^ TAMs remain independent prognostic factors for freedom from treatment failure (PD-L1^+^CD68^+^/CD68^+^, HR = 2.63, 95% CI 1.17–5.88, *p* = 0.019; IDO-1^+^CD68^+^/CD68^+^, HR = 2.48, 95% CI 1.03–5.95, *p* = 0.042). In contrast, proportions of PD-L1^+^ tumor cells, all TAMs or PD-L1^−^ and IDO-1^−^ TAMs are not associated with outcome. The findings implicate that adverse prognostic impact of TAMs is checkpoint-dependent in cHL.

## 1. Introduction

Classical Hodgkin lymphoma (cHL) is characterized by unique cell composition of the tumor, where typically low numbers of neoplastic Hodgkin and Reed–Sternberg cells (HRS cells) are surrounded by abundant reactive inflammatory cells [1]. HRS cells express numerous immunoregulatory proteins that can shape the microenvironment and allow tumor cells to escape immune surveillance [2,3]. One example of this is immune evasion via the programmed cell death-1 (PD-1) pathway due to the genetic/genomic alterations of chromosome 9p24.1, which leads to increased expression of PD-1 ligand 1 and 2 (PD-L1 and PD-L2) in HRS cells [4] and T-cell exhaustion [5], and finally translates to poor survival in patients treated with chemotherapy [4].

Apart from HRS cells, PD-L1 and PD-L2 are expressed in the tumor microenvironment (TME) [6], and PD-L1 particularly in tumor-associated macrophages (TAMs), which are located physically in the vicinity of PD-L1^+^ HRS cells [7]. Macrophages also express other immunosuppressive molecules, such as indoleamine 2,3-dioxygenase (IDO-1), an enzyme responsible for catabolizing tryptophan into kynurenine metabolites [8]. IDO-1 activity can lead to the inhibition of T-cells by suppression of effector T-cell function and activation of regulatory T-cells [8,9], which is a potential mechanism enabling tumor cells to avoid host immune response. In cHL, both an increased proportion of PD-L1^+^ leukocytes [10] and IDO expression in the TME cells in nodular sclerosis (NS) subtype [11] have been associated with inferior overall survival.

Several studies have demonstrated that high numbers of either CD68^+^ or CD163^+^ TAMs translate to unfavorable survival in cHL [12,13,14,15,16,17]. There are also studies, however, in which this association has not been confirmed [18,19,20,21]. The purpose of this study is to digitally quantify TAM abundance and to characterize TAM immunophenotypes with regard to PD-L1 and IDO-1 expression from diagnostic cHL tumor samples, as well as to associate the findings with clinical characteristics and survival.

## 2. Results

### 2.1. Patient Characteristics and Outcome

The main multiplex immunohistochemistry (mIHC) cohort consisted of 130 cHL patients whose demographics and outcome are described in Table 1. The median age of the patients was 29 years, and the majority represented the NS subtype, had advanced stage, were negative for Epstein–Barr virus (EBV), had low International Prognostic Score (IPS), and were treated with doxorubicin, bleomycin, vinblastine and dacarbazine (ABVD), followed by radiotherapy. During the follow-up time, 28 patients relapsed and 10 died, six of them from cHL. The 5-year freedom from treatment failure (FFTF), disease-specific survival (DSS), and overall survival (OS) were 80%, 94% and 91%, respectively.

### 2.2. Association of IDO-1, PD-L1, CD68, CD163 Gene Expressions with Outcome

First, for screening purposes we measured mRNA expression levels of both macrophage markers (*CD68* and *CD163*) and selected checkpoint molecules *CD274* (gene encoding PD-L1), as well as *IDO-1* from 88 diagnostic cHL samples. Then, we examined whether their gene expressions in the tumor tissue correlated with each other. *CD274* expression correlated positively with *CD68* (ρ = 0.688, *p* < 0.001) and to a lesser extent with *CD163* expression (ρ = 0.362, *p* = 0.001). *IDO-1* expression correlated with *CD68* expression (ρ = 0.386, *p* < 0.001), whereas no correlation with *CD163* expression was found. Expressions of *CD68* and *CD163* correlated with each other (ρ = 0.549, *p* < 0.001) (Figure S1a). Interestingly, when analyzed as continuous variables, high *CD274* and *IDO-1* expression translated to poor FFTF, and high *IDO-1* expression also translated to poor DSS and OS. In addition, high *CD68* expression correlated with inferior OS, whereas *CD163* expression was not associated with outcome (Table 2).

### 2.3. High Number of PD-L1^+^ and IDO-1^+^ Cells Translates to Inferior Outcome

**Figure 1:**
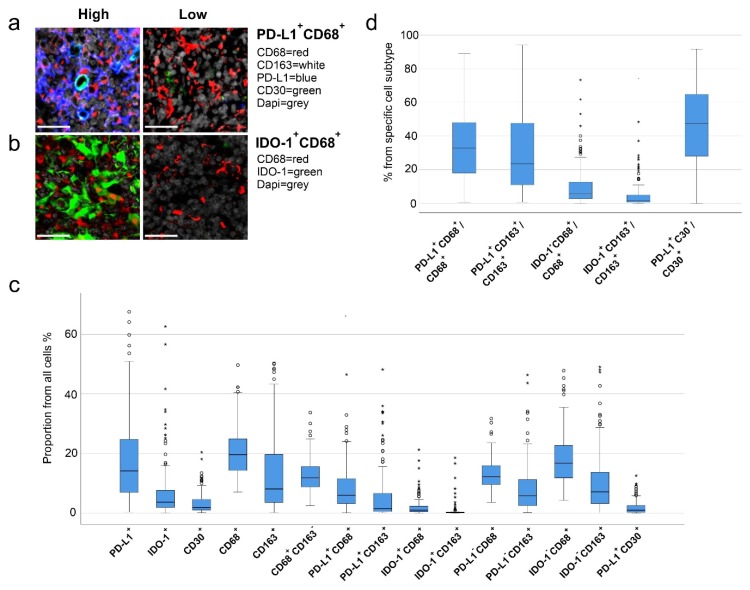
Immunophenotypes of different cells. Representative images of (**a**) PD-L1^+^CD68^+^ and (**b**) IDO-1^+^CD68^+^ high and low cell proportions from all cells (scale bars 30 µm). (**c**) Boxplots representing proportions of different cell types from all cells. (**d**) Boxplots representing proportions of PD-L1^+^ and IDO-1^+^ tumor-associated macrophages (TAMs) from all TAMs and PD-L1^+^CD30^+^ cells from all CD30^+^ cells.

To further examine the expression of PD-L1 and IDO-1 proteins in the tumor tissue, and particularly in TAMs, we profiled the cellular immunophenotypes with antibody-based mIHC. As a general marker of TAMs, we used CD68, whereas subpopulations of TAMs were defined by the presence or absence of CD163, PD-L1 and IDO-1 (Figure 1a,b). There was a good correlation between the gene expression and the mIHC data. The proportions of CD68^+^ cells, CD163^+^ cells, IDO-1^+^ cells, and PD-L1^+^ cells in the mIHC analysis correlated with the gene expression of *CD68* (ρ = 0.681, *p* < 0.001), *CD163* (ρ = 0.764, *p* < 0.001), *CD274* (ρ = 0.688, *p* < 0.001) and *IDO-1* (ρ = 0.762, *p* < 0.001), respectively (Figure S1b). In addition, in the mIHC analysis the quantities of PD-L1^+^ and IDO-1^+^ cells correlated with CD68^+^ cells (PD-L1^+^, ρ = 0.691, *p* < 0.001; IDO-1^+^, ρ = 0.196, *p* = 0.025) and CD163^+^ cells (PD-L1^+^, ρ = 0.374, *p* < 0.001; IDO-1^+^, ρ = 0.206, *p* = 0.019). Furthermore, CD68^+^ and CD163^+^ cells correlated with each other (ρ = 0.626, *p* < 0.001) (Figure S1c). Finally, IDO-1^+^ and PD-L1^+^ macrophages correlated with interferon γ gene expression (Table S1). The proportions of distinct cell subsets in the cHL tissue are shown in Figure 1c. CD68^+^ and CD163^+^ TAM contents, as well as the PD-L1^+^ and IDO-1^+^ cells contents from all cells, showed great variation between the samples (CD68^+^ TAMs, median 20%, range 7.0–50%; CD163^+^ TAMs, median 8.6%, range 0.2–50%; PD-L1^+^ cells, median 14%, range 0.1–68% and IDO-1^+^ cells, median 3.7%, range 0–63%; Figure 1c). Consistent with the gene expression data, high PD-L1^+^ and IDO-1^+^ cell contents translated to poor FFTF, DSS and OS when analyzed as continuous variables, whereas the proportions of either CD68^+^, CD163^+^, or CD68^+^CD163^−^ TAMs of all cells did not correlate with survival (Table 3).

### 2.4. High Proportions of PD-L1^+^ and IDO-1^+^ TAMs Translate to Inferior Outcome

**Figure 2:**
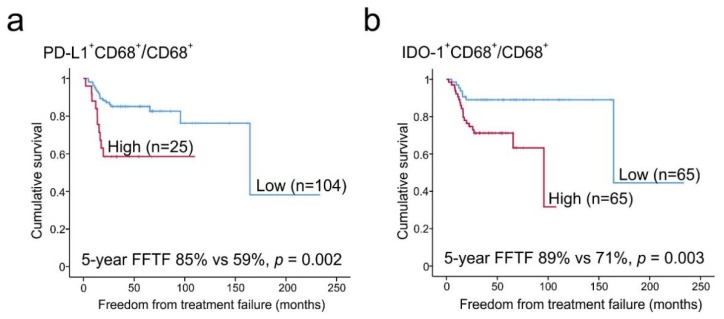
Association of the macrophage immunophenotypes with FFTF. Kaplan–Meier estimates for FFTF according to (**a**) PD-L1^+^CD68^+^/CD68^+^ cell ratio (cut-off highest fifth, 50%) and (**b**) IDO-1^+^CD68^+^/CD68^+^ cell ratio (cut-off median, 5.5%) dividing the patients into high and low cell ratio subgroups.

We further observed that a significant amount of PD-L1 (median 45%, range 15–85%) and IDO-1 (median 22%, range 0–62%) was expressed in macrophages (Figure 1c). Of the CD68^+^ and CD163^+^ M2-like TAMs, 32% (range 0.2–89%) and 22% (range 0.1–94%) expressed PD-L1, whereas fewer CD68^+^ (median 5.5%, range 0–73%) and CD163^+^ TAMs (median 1.4%, range 0–74%) were characterized as IDO-1^+^ (Figure 1d). Both high proportions of PD-L1^+^ or IDO-1^+^ macrophages from all cells, and high proportions of PD-L1^+^ or IDO-1^+^ macrophages from all macrophages (high PD-L1^+^CD68^+^/CD68^+^, PD-L1^+^CD163^+^/CD163^+^, IDO-1^+^CD68^+^/CD68^+^ and IDO-1^+^CD163^+^/CD163^+^ cell ratio) translated to inferior survival when analyzed as continuous variables (Table 3). In contrast, neither the ratio of PD-L1^-^ nor IDO-1^-^ macrophages from all cells were associated with an outcome. Furthermore, based on the proportions of PD-L1^+^ and IDO-1^+^ macrophages from all macrophages, patients were divided into two subgroups with low and high PD-L1^+^CD68^+^/CD68^+^ and IDO-1^+^CD68^+^/CD68^+^ ratios (Figure 2). According to Kaplan–Meier estimates, the 5-year FFTF rates were significantly worse for the patients with high ratio of PD-L1^+^ TAMs (59% vs. 85%, *p* = 0.002) and IDO-1^+^ TAMs (71% vs. 89%, *p* = 0.003) from all macrophages in comparison to patients with low ratios. When the distribution of the baseline characteristics was compared between the high and low subgroups, no significant differences in gender, age and IPS were observed (Table 4). However, patients with high PD-L1^+^CD68^+^/CD68^+^ or IDO-1^+^CD68^+^/CD68^+^ proportions more frequently had other cHL subtypes than NS, advanced than limited stage disease and were EBV positive.

### 2.5. PD-L1 Expression in HRS Cells

We also examined association of CD30^+^ HRS cells with PD-L1 positivity. As expected, the median proportion of CD30^+^ HRS cells from the whole tumor cellularity was low (median 1.8%, range 0.06–20%), and about half of CD30^+^ HRS cells were PD-L1^+^ (median 47%, range 0–92%) (Figure 1c). The proportion of PD-L1^+^ HRS cells (PD-L1^+^CD30^+^/CD30^+^) correlated with the proportion of PD-L1^+^ macrophages (PD-L1^+^CD68^+^/CD68^+^; ρ = 0.479, *p* < 0.001). While high CD30^+^ cell content in the tumor tissue translated to inferior DSS, neither the proportion of PD-L1^+^CD30^+^ cells from all cells, nor the PD-L1^+^CD30^+^/CD30^+^ cell ratio correlated with survival (Table 3).

### 2.6. Prognostic Impact of PD-L1^+^ and IDO-1^+^ TAM Proportions

To further assess the prognostic value of high and low PD-L1^+^ and IDO-1^+^ TAM ratios with clinical risk factors, Cox regression analyses were performed with categorical variables (Table 5). Besides subgroups of patients with high PD-L1^+^CD68^+^/CD68^+^ and IDO-1^+^CD68^+^/CD68^+^ cell ratios, age (≥60 years) and stage (IIB-IV) had adverse prognostic impact on FFTF in our mIHC cohort. High PD-L1^+^CD68^+^/CD68^+^ cell ratio and high age (≥60 years) also had adverse prognostic impact on DSS and OS, whereas EBV positivity and high IPS (4–7) were associated only with poor OS. Gender or cHL subtype did not have any association with survival. High PD-L1^+^CD68^+^/CD68^+^ ratio remained as an adverse prognostic factor for FFTF, DSS and OS when adjusted for cHL subtype, and also for FFTF and DSS when adjusted for stage or EBV status, whereas the association with OS remained significant only in the EBV negative cases (EBV-, HR = 7.687, (95% CI 1.067–55.362), *p* = 0.043; EBV+, HR = 1.860 (95% CI 0.306–11.296), *p* = 0.500) and was more evident in the patients with advanced than limited stage (Stage IIB-IV, HR = 3.824 (95% CI 0.935–15.636), *p* = 0.062; stage I-IIA, HR = 0.043 (95% CI 0.000–2.971 ×1011), *p* = 0.835). Furthermore, when IDO-1^+^CD68^+^/CD68^+^ content was adjusted for stage, cHL subtype or EBV status, a high ratio remained as an adverse prognostic factor for FFTF. In multivariate analysis, both PD-L1^+^CD68^+^/CD68^+^ ratio and IDO-1^+^CD68^+^/CD68^+^ ratio predicted FFTF independently of age and stage (Table 6).

## 3. Discussion

The aim of our study was to quantify and characterize immunophenotypes of TAMs and investigate whether PD-L1^+^ and IDO-1^+^ TAMs have prognostic value in primary cHL patients treated with standard chemo- and radiotherapy. First, we observed that high gene expression levels of PD-L1 and IDO-1 were associated with poor survival. A similar correlation was observed at the protein level. Given the accentuated expression of PD-L1 and IDO-1 in TAMs, high proportions of PD-L1^+^ TAMs and IDO-1^+^ TAMs also translated to inferior outcome. These survival associations were seen within both overall CD68^+^ TAMs and in CD163^+^ putative M2-like TAMs, suggesting subtype-independent association, although M2-like alternatively activated macrophages have been considered to be pro-tumoral [3] and promote immunosuppression [22]. On the contrary, in our study population, neither the proportion of macrophages (CD68^+^ or CD163^+^), nor the PD-L1^−^ or IDO-1^−^ TAMs translated to survival. These results indicate that only distinct TAM immunophenotypes mediate significant adverse impact on survival. 

Our data emphasize the diversity of the TME, since the proportions of different cell types varied significantly between the patients, as did the cell ratios of PD-L1^+^ and IDO-1^+^ macrophages from all macrophages. Furthermore, despite the finding that only a very small proportion of CD163^+^ TAMs were IDO-1^+^ (median 1.4%), these few cells had a notably adverse impact on the treatment outcome, highlighting the importance of the subgroup of macrophages as immunomodulators in the TME. Furthermore, considering the variation of checkpoint molecule expression on TAMs, our findings may potentially provide explanation for the opposite results in previous studies, which have investigated the prognostic impact of CD68^+^ and/or CD163^+^ macrophages [15]. 

Although the overall proportion of PD-L1^+^ cells was associated with inferior outcome, neither the proportion of PD-L1^+^CD30^+^ cells nor the ratio of PD-L1^+^CD30^+^/CD30^+^ cells translated to inferior survival. This finding is important by demonstrating that the adverse prognostic impact of PD-L1 expression is TME-dependent, and, particularly, TAM-dependent. Interestingly, two previous studies also showed that neither the expression of PD-L1 nor PD-L2 on HRS cells is associated with outcome [10,23]. Unfortunately, we could not address the same question with IDO-1, because our mIHC antibody panel design did not allow analysis of IDO-1 and CD30 expression simultaneously. However, it has been observed previously that IDO-1 is not expressed in HRS cells [11]. Together with previous results, our data imply that prognostic impact of IDO-1 expression is also derived from the TME and TAMs. Whether IDO-1 and PD-L1 are expressed on the same macrophages and whether those macrophages have even more remarkable association with outcome remains unanswered in the framework of this study. However, our results propose that PD-L1^+^ TAMs are more abundant than IDO-1^+^ TAMs. The overall proportion of PD-L1^+^ cells is also higher than IDO-1^+^ cells in the tumor tissue, indicating variation between the expression of these two molecules on macrophages. 

We have previously demonstrated the importance of PD-L1^+^ TAMs on survival in patients with primary testicular lymphoma [24]. To our knowledge, however, this is the first study in cHL to show that high proportions of IDO-1 and PD-L1 expressing TAMs from all TAMs predict worse FFTF independently from clinical risk factors. This was evident regardless of the EBV status, and in contrast to previous studies showing that survival association of TAMs is limited to EBV negative cases [16,25]. Furthermore, in this study, high ratios of PD-L1^+^CD68^+^/CD68^+^ and IDO-1^+^CD68^+^/CD68^+^ were associated with EBV positivity, which is in line with previous studies showing higher expression of macrophages [13,26], PD-L1 [4] and IDO [11] in EBV positive rather than negative cases. The correlation of PD-L1^+^ and IDO-1^+^ TAMs with interferon γ gene expression in turn suggests an interaction between TAMs and cytotoxic T lymphocytes. 

The mechanism for immune evasion via IDO-1^+^ and PD-L1^+^ macrophages remains unclear, and further studies are needed to resolve this question. However, PD-L1^+^ TAMs have been shown to be in contact both with PD-1^+^CD4^+^ T-cells and PD-1^+^CD8^+^ T-cells in the close vicinity of HRS cells [7], possibly implying that TAMs interact with PD-1^+^ T-cells in promoting immunosuppression. Interestingly, suppression of PD-1^+^ natural killer (NK) cells has also been recently shown to occur via PD-L1 expressing CD163^+^ TAMs, and more prominently in cHL than in diffuse large cell lymphoma (DLBCL) [27]. 

PD-1 blockade with monoclonal antibodies has demonstrated promising response rates (65–87%) and long-term remissions in a subgroup of poor prognosis patients with relapsed and refractory cHL [28,29,30,31]. Currently, there are several ongoing clinical trials investigating PD-1 blockade alone as a first-line treatment or in combination with chemotherapy or brentuximab vedotin [32,33,34]. Interestingly, it has previously been suggested that therapies affecting the PD-1 pathway may also function through macrophages [35]. IDO-1 inhibitors are also under investigation in clinical trials alone and in combination with other therapies in different advanced malignancies, including lymphomas [8,36]. Our results suggest that IDO-1^+^ and PD-L1^+^ TAMs could be potential targets for novel immunotherapies. They might also be useful biomarkers to stratify treatments in cHL. The patients with high proportions of IDO-1^+^ or PD-L1^+^ macrophages at the time of diagnosis may define subgroups that particularly benefit from PD-1 and/or IDO-1 blockade.

In this study we have demonstrated that high proportions of PD-L1⁺ and IDO-1^+^ TAMs are both associated with unfavorable outcomes in cHL patients treated with standard chemotherapy. The results should be confirmed prospectively in an independent cohort of cHL patients. Nevertheless, they provide rationale for studying PD-1 and IDO-1 inhibitors in combination with standard chemotherapy for patients with high PD-L1⁺ and IDO-1^+^ TAM content in their tumor tissue.

## 4. Materials and Methods

### 4.1. Patients and Samples

The study material included clinical data and diagnostic formalin-fixed paraffin-embedded (FFPE) tumor tissue samples from the patients with primary cHL. All patients were diagnosed between the years 1993–2012 and were treated or followed in Helsinki University Hospital. Patients and corresponding clinical data were retrospectively extracted from electronic and/or paper-based medical records. Gene expression data from 88 patients enriched in elderly and relapsed/refractory (R/R) cases were used for screening. The main study cohort consisted of 130 patients, who were selected based on the availability of representative tumor tissue for tissue microarray (TMA) and which was named as “mIHC cohort”. Seventy-eight patients were overlapping between the gene expression dataset and the mIHC cohort. 

Detailed description of staging procedures, response evaluation and treatment is provided in the Supplementary Materials.

Patient data were handled according to Good Scientific Practice (GSP) Guidelines. The study was approved by the Ethics Committee in Helsinki University Hospital (Finland; HUS/1230/2017), and by the Finnish National Authority for Medicolegal Affairs (9505/06.01.03.01/2013), which waived the requirement to obtain informed consent.

### 4.2. Gene Expression Analysis

Gene expression levels of macrophage markers *CD68* and *CD163*, *INFG (*gene encoding interferon *γ)*, and immunosuppressive molecules *CD274* (gene encoding PD-L1) and *IDO-1* were measured from 88 samples using digital gene expression analysis with NanoString nCounter (NanoString Technologies, Seattle, WA, USA) [37].

### 4.3. Multiplex Immunohistochemistry

TMA was constructed from one to six replicate spots of the same FFPE tumor tissue. Core selection on the TMA was based on the evaluation of a hematopathologist. TMA sections were stained with panels of primary antibodies for CD68, CD163, PD-L1, IDO-1 and CD30. CD68 was used as universal macrophage marker, CD163 as marker for M2-polarized macrophages, CD30 as a marker to recognize HRS-cells, and PD-L1 and IDO-1 for immunosuppression. The quantities of different immunophenotypes were counted as proportion from all cells or from specific cells on the whole TMA spot. The mean value of the cell proportions from the same tissue samples replicate spots were used. The mIHC analysis was performed digitally using the open-source platform CellProfiler [38]. A description of mIHC method is given in the Supplementary Materials. Antibodies for CD68, CD163 and IDO-1 were included in two panels, and in the analysis the mean values of the cell proportions from these two separate panels were used. The correlations were high between the panels for cell proportions of CD68^+^ (ρ = 0.736, *p* < 0.001), CD163^+^ (ρ = 0.960, *p* < 0.001) and IDO-1^+^ cells (ρ = 0.939, *p* < 0.001), respectively, emphasizing the reliability and repeatability of the method.

### 4.4. Determination of Epstein-Barr Virus Status

EBV status was determined as either negative or positive using Epstein–Barr virus encoded RNA (EBER) in situ hybridization (ISH) for the TMAs. Cases with positive nuclear staining of HRS-cells were qualified as EBV positive.

### 4.5. Statistical Analysis

FFTF was defined as the time between the date of the diagnosis and disease progression, including progression during primary therapy and later relapses. OS was defined as time between the date of the diagnosis and death from any cause, and DSS as the time between the date of the diagnosis and death due to cHL. 

Statistical analyses were processed with IBM SPSS v.25.0 (IBM, Armonk, NY, USA). The chi-square test was used to assess differences in the frequency of individual prognostic factors. Univariate and multivariate analyses were performed according to Cox proportional hazards regression model. Survival rates were estimated with the Kaplan–Meier method and the differences were compared using a log-rank test. In Kaplan–Meier analysis the highest fifth (50%) and the median (5.5%) values were used as cut-off levels to divide the patients to high and low PD-L1^+^CD68^+^/CD68^+^ and IDO-1^+^CD68^+^/CD68^+^ cell ratio subgroups, respectively.

Correlation analyses were performed with Spearman rank analysis. A level of probability below 0.05 was considered significant. All comparisons were two-tailed.

## 5. Conclusions

Earlier studies have highlighted the important role of TAMs in the pathogenesis of cHL, as their high proportion has been associated with inferior outcomes. In this study we demonstrate for the first time that the adverse prognostic impact of TAMs on survival is checkpoint dependent, and more specifically PD-L1 and IDO-1 expression-dependent; high content of PD-L1^+^ and IDO-1^+^ TAMs in pretreatment tumor samples translates to poor survival in patients treated with standard chemo- and radiotherapy. Our findings indicate that PD-L1^+^ and IDO-1^+^ TAMs play important roles as immunomodulators in the TME and are potential new biomarkers for treatment stratification as well as potential new targets for novel immunotherapies. 

## Figures and Tables

**Table 1:** Patient demographics and outcome.

Characteristic	*n* = 130 (%)
Median follow-up time, months (range)	55 (7–229)
Age (years)	
Median (range)	29 (16–83)
<60	116 (89)
≥60	14 (11)
Sex	
Male	59 (45)
Female	71 (55)
Histologic subtype	
Nodular sclerosis	102 (78)
Mixed cellularity	21 (16)
Lymphocyte-rich	6 (5)
Unclassified cHL	1 (1)
Stage	
I-IIA	56 (43)
IIB-IV	73 (56)
NA	1 (1)
EBV status	
Negative	89 (69)
Positive	34 (26)
NA	7 (5)
IPS	
0–3	86 (66)
4–7	7 (5)
NA	37 (29)
Treatment	
ABVD	41 (32)
ABVD + radiotherapy	70 (53)
BEACOPPesc	5 (4)
BEACOPPesc + radiotherapy	4 (3)
ABVD + BEACOPPesc	4 (3)
CHOP	4 (3)
Other	2 (2)
Radiotherapy *	77 (59)
Relapses	28 (22)
Deaths	10 (8)
cHL related deaths	6 (60)
5-year FFTF	80%
5-year DSS	94%
5-year OS	91%

* Including chemotherapy and radiotherapy and radiotherapy only. ABVD, doxorubicin, bleomycin, vinblastine, dacarbazine; BEACOPPesc, bleomycin, etoposide, doxorubicin, cyclophosphamide, vincristine, procarbazine, prednisone in escalated dose; CHOP, cyclophosphamide, doxorubicin, vincristine, prednisone; NA, not assigned; EBV, Epstein–Barr virus; IPS, International Prognostic Score; FFTF, freedom from treatment failure; DSS, disease-specific survival; OS, overall survival.

**Table 2:** Cox regression analysis as continuous variable at univariate level showing association of gene expression levels with FFTF, DSS and OS.

Gene Symbol	FFTF			DSS			OS		
	HR	95% CI	*p*	HR	95% CI	*p*	HR	95% CI	*p*
*CD274* (PD-L1)	**1.607**	**1.027–2.513**	**0.038**	2.091	0.871–5.024	0.099	1.791	0.816–3.931	0.146
*IDO-1*	**1.465**	**1.069–2.009**	**0.018**	**2.234**	**1.327** **–** **3.762**	**0.003**	**2.107**	**1.311** **–** **3.388**	**0.002**
*CD68*	1.256	0.768–2.054	0.364	2.319	0.923–5.826	0.074	**2.405**	**1.056** **–** **5.475**	**0.037**
*CD163*	0.895	0.691–1.161	0.404	1.217	0.771–1.923	0.399	1.364	0.908–2.050	0.135

HR, hazard ratio; CI, confidence interval; FFTF, freedom from treatment failure; DSS, disease-specific survival; OS, overall survival. Boldface font indicates statistical significance (*p* < 0.05).

**Table 3:** Cox regression analysis as continuous variables at univariate level showing association of cell immunophenotypes with FFTF, DSS and OS.

Cell Immunophenotype (proportion from all cells)	FFTF			DSS			OS		
	HR	95% CI	*p*	HR	95% CI	*p*	HR	95% CI	*p*
PD-L1^+^	**1.027**	**1.003–1.051**	**0.025**	**1.069**	**1.018–1.123**	**0.007**	**1.054**	**1.012–1.098**	**0.011**
IDO-1^+^	**1.048**	**1.020–1.076**	**0.001**	**1.082**	**1.042–1.123**	**<0.001**	**1.074**	**1.039–1.111**	**<0.001**
CD30^+^	1.059	0.967–1.160	0.217	**1.147**	**1.001–1.315**	**0.049**	1.087	0.949–1.245	0.227
CD68^+^	1.012	0.970–1.055	0.594	1.045	0.956–1.143	0.331	1.061	0.989–1.139	0.098
CD163^+^	1.009	0.981–1.037	0.541	1.035	0.981–1.091	0.206	1.041	0.997–1.086	0.066
CD68^+^CD163^-^	1.022	0.959–1.088	0.506	1.009	0.877–1.161	0.899	0.994	0.883–1.118	0.914
PD-L1^+^CD68^+^	**1.042**	**1.002–1.084**	**0.040**	**1.109**	**1.031–1.194**	**0.006**	**1.093**	**1.026–1.164**	**0.006**
PD-L1^+^CD163^+^	1.029	0.993–1.066	0.114	**1.099**	**1.030–1.172**	**0.004**	**1.088**	**1.031–1.148**	**0.002**
IDO-1^+^CD68^+^	**1.107**	**1.021–1.201**	**0.014**	**1.235**	**1.107–1.378**	**<0.001**	**1.221**	**1.105–1.348**	**<0.001**
IDO-1^+^CD163^+^	**1.181**	**1.070–1.304**	**0.001**	**1.319**	**1.163–1.495**	**<0.001**	**1.290**	**1.151–1.447**	**<0.001**
PD-L1^−^CD68^+^	0.980	0.914–1.051	0.576	0.918	0.763–1.104	0.363	0.987	0.872–1.117	0.837
PD-L1^−^CD163^+^	0.991	0.947–1.037	0.703	0.962	0.848–1.091	0.543	1.001	0.940–1.087	0.770
IDO-1^−^CD68^+^	0.976	0.928–1.025	0.330	0.884	0.755–1.036	0.129	0.982	0.898–1.074	0.690
IDO-1^−^CD163^+^	0.993	0.956–1.032	0.732	0.987	0.902–1.080	0.775	1.015	0.960–1.074	0.595
PD-L1^+^CD30^+^	1.063	0.922–1.225	0.402	1.192	0.928–1.532	0.169	1.093	0.854–1.401	0.480
Cell immunophenotype (proportion from specific cell subtype)	HR	95% CI	*p*	HR	95% CI	*p*	HR	95% CI	*p*
PD-L1^+^CD68^+^/CD68^+^	**1.021**	**1.003–1.039**	**0.024**	**1.047**	**1.005–1.090**	**0.027**	**1.034**	**1.001–1.068**	**0.042**
PD-L1⁺CD163^+^/CD163^+^	**1.020**	**1.006–1.035**	**0.005**	**1.038**	**1.005–1.072**	**0.022**	**1.028**	**1.002–1.054**	**0.036**
IDO-1^+^CD68⁺/CD68^+^	**1.032**	**1.009–1.057**	**0.007**	**1.066**	**1.031–1.102**	**<0.001**	**1.059**	**1.028–1.091**	**<0.001**
IDO-1^+^CD163^+^/CD163^+^	**1.040**	**1.015–1.066**	**0.002**	**1.062**	**1.030–1.094**	**<0.001**	**1.057**	**1.028–1.087**	**<0.001**
PD-L1^+^CD30^+^/CD30^+^	1.008	0.992–1.024	0.323	1.016	0.981–1.053	0.369	1.008	0.981–1.036	0.555

HR, hazard ratio; CI, confidence interval; FFTF, freedom from treatment failure; DSS, disease-specific survival; OS, overall survival. Boldface font indicates statistical significance (*p* < 0.05).

**Table 4:** Distribution of baseline characteristics between high and low cell ratio of PD-L1^+^CD68^+^/CD68^+^ and IDO-1^+^CD68^+^/CD68^+^.

Characteristic	PD-L1^+^CD68^+^/CD68^+^			IDO-1^+^CD68^+^/CD68^+^		
	Low	High	*p*	Low	High	*p*
Number of Patients (%)	104	25		65	65	
Sex						
Male	45 (43)	13 (52)	0.431	29 (45)	30 (46)	0.86
Female	59 (57)	12 (48)		36 (55)	35 (54)	
Age (years)			0.837			
<60	93 (89)	22 (88)		60 (92)	56 (86)	0.258
≥60	11 (11)	3 (12)		5 (8)	9 (14)	
Subtype						
NS	86 (83)	16 (64)	**0.039**	57 (88)	45 (69)	**0.01**
Other *	8 (17)	9 (36)		8 (12)	20 (31)	
Stage						
I-IIA	49 (48)	6 (24)	**0.033**	34 (53)	22 (34)	**0.027**
IIB-IV	54 (52)	19 (76)		30 (47)	43 (66)	
EBV status						
Negative	75 (76.5)	13 (54)	**0.029**	48 (81)	23 (64)	**0.032**
Positive	23 (23.5)	11 (46)		11 (19)	41 (36)	
IPS						
0–3	66 (92)	20 (95)	0.585	42 (93)	44 (92)	0.761
4–7	6 (8)	1 (5)		3 (7)	4 (8)	

* Other: Mixed cellularity (MC) + other/unclassified cHL. NS, nodular sclerosis; EBV, Epstein–Barr virus; IPS, International Prognostic Score. Boldface font indicates statistical significance (*p* < 0.05).

**Table 5:** Cox regression analysis as categorical variables at univariate level showing association of PD-L1^+^CD68^+^/CD68^+^ cell ratio, IDO-1^+^CD68^+^/CD68^+^ cell ratio and clinical characteristics of mIHC cohort with FFTF, DSS and OS.

Characteristic	FFTF			DSS			OS		
	HR	95% CI	*p*	HR	95% CI	*p*	HR	95% CI	*p*
PD-L1^+^CD68^+^/CD68^+^ (high)	**3.222**	**1.46–7.09**	**0.004**	**11.958**	**2.15–66.63**	**0.005**	**4.646**	**1.23–17.54**	**0.023**
IDO-1^+^CD68^+^/CD68^+^ (high)	**3.537**	**1.47–8.50**	**0.005**	6.050	0.70–52.21	0.102	4.237	0.88–20.52	0.073
PD-L1^+^CD68^+^/CD68^+^ (high) Stage adjusted (I-IIA vs. IIB-IV)	**2.525**	**1.12–5.68**	**0.025**	**8.243**	**1.46–46.64**	**0.017**	3.348	0.87–12.86	0.078
IDO-1^+^CD68^+^/CD68^+^ (high) Stage adjusted (I-IIA vs. IIB-IV)	**2.586**	**1.08–6.17**	**0.032**	4.154	0.48–35.77	0.195	3.301	0.67–16.38	0.144
PD-L1^+^CD68^+^/CD68^+^ (high) Subtype adjusted (NS vs. others)	**3.455**	**1.54–7.75**	**0.003**	**11.123**	**1.93–64.09**	**0.007**	**4.202**	**1.08–16.36**	**0.038**
IDO-1^+^CD68^+^/CD68^+^ (high) Subtype adjusted (NS vs. others)	**3.419**	**1.41–8.32**	**0.007**	5.711	0.65–51.57	0.117	3.957	0.79–19.86	0.095
PD-L1^+^CD68^+^/CD68^+^ (high) EBV status adjusted (neg vs. pos)	**3.715**	**1.64–8.41**	**0.002**	**11.071**	**1.91–64.06**	**0.007**	3.450	0.88–13.50	0.075
IDO-1^+^CD68^+^/CD68^+^ (high) EBV status adjusted (neg vs. pos)	**3.937**	**1.54–10.06**	**0.004**	5.390	0.61–47.52	0.129	3.237	0.65–16.03	0.150
Age (≥60y)	**3.799**	**1.60–9.00**	**0.002**	**12.708**	**2.46–65.55**	**0.002**	**16.516**	**4.30–63.37**	**<0.001**
Stage (IIB-IV)	**7.791**	**2.35–25.82**	**0.001**	55.058	0.07–41007.16	0.235	6.631	0.83–53.06	0.075
Female	0.676	0.32–1.42	0.303	0.746	0.15–3.71	0.721	0.596	0.16–2.23	0.442
EBV-status (positive)	0.811	0.33–2.02	0.653	1.608	0.29–8.84	0.585	**4.100**	**1.09–15.38**	**0.036**
IPS (4–7)	1.157	0.27–4.95	0.854	4.603	0.48–44.65	0.188	**9.725**	**1.61–58.77**	**0.013**
Other cHL subtype than NS	1.106	0.44–2.76	0.829	2.527	0.45–14.35	0.295	2.582	0.63–10.61	0.188

IPS, International Prognostic Score; EBV, Epstein–Barr virus; NS, nodular sclerosis; HR, hazard ratio; CI, confidence interval; FFTF, freedom from treatment failure; DSS, disease-specific survival; OS, overall survival. Boldface font indicates statistical significance (*p* < 0.05).

**Table 6:** Cox regression analysis at multivariate level showing independent association of PD-L1^+^CD68^+^/CD68^+^ or IDO-1^+^CD68^+^/CD68^+^ cell ratio, stage and age with FFTF.

Risk Factor	HR	95% CI	*p*
PD-L1^+^CD68^+^/CD68^+^ (high)	**2.625**	**1.173–5.876**	**0.019**
Stage (IIB-IV)	**5.834**	**1.720–19.786**	**0.005**
Age (≥60 years)	**2.631**	**1.095–6.319**	**0.031**
IDO-1^+^CD68^+^/CD68^+^ (high)	**2.480**	**1.034–5.947**	**0.042**
Stage (IIB-IV)	**5.799**	**1.705–19.717**	**0.005**
Age (≥60 years)	2.221	0.921–5.356	0.076

HR, hazard ratio; CI, confidence interval; FFTF, freedom from treatment failure. Boldface font indicates statistical significance (*p* < 0.05).

## Supplementary Materials

The following are available online at https://www.mdpi.com/2072-6694/12/4/877/s1. Figure S1: Correlation by Spearman rank analysis between gene and protein expression levels of macrophage markers, CD274/PD-L1 and IDO-1, Table S1: Correlations by spearman rank analysis between interferon gamma gene expression levels and different cell proportions in the mIHC analysis.
