# Supplementary material for: Prognostic Impact of Tumor-Associated Macrophages on Survival Is Checkpoint Dependent in Classical Hodgkin Lymphoma
Source: Cancers (Basel). 2020 Apr 4;12(4):877. doi: 10.3390/cancers12040877 (PMC7225916; doi:10.3390/cancers12040877)
Supplement: Supplementary file 1 [file cancers-12-00877-s001.pdf]

Supplementary Material

Prognostic impact of tumor-associated macrophages on survival is checkpoint dependent in classical Hodgkin lymphoma

Kristiina Karihtala, Suvi-Katri Leivonen, Oscar Brück, Marja-Liisa Karjalainen-Lindsberg, Satu Mustjoki, Teijo Pellinen and Sirpa Leppä

Supplementary Methods

Data collection, disease staging, response evaluation and treatment

Clinical data was collected retrospectively from medical records and formalin-fixed paraffin-embedded (FFPE) tumor tissues from pathology archives. Staging was carried out by Ann Arbor classification and treatment was according to the current national guidelines. In general, the combined modality treatment, which was used in limited disease, consisted of 2-4 cycles of chemotherapy based on clinical risk factors followed by involved field radiotherapy. In advanced disease, the treatment consisted of 6-8 cycles of chemotherapy and additional radiotherapy, when necessary. The primary chemotherapy was mainly ABVD (doxorubicin, bleomycin, vinblastine, dacarbazine) and less frequently in advanced stage high risk patients BEACOPPescalated (bleomycin, etoposide, doxorubicin, cyclophosphamide, vincristine, procarbazine, prednisone in escalated dose). In elderly patients not eligible for ABVD, CHOP (cyclophosphamide, doxorubicin, vincristine, prednisone) was also used [1]. Positron emission tomography-computed tomography (PET-CT) was not at that time routinely used for staging and response evaluation. For the eligible patients with relapsed/refractory (R/R) disease, the secondary treatment consisted of high dose chemotherapy followed by autologous stem cell transplantation.

Multiplex immunohistochemistry

Antibodies CD68 (Ab955, Abcam, Cambridge, United Kingdom), CD163 (ab188571, Abcam, Cambridge, United Kingdom), PD-L1 (13684, CST, Danvers, MA, United States) IDO-1 (86630, CST, Danvers, United States) and CD30 (130M-94, Cell Marque, Rocklin, CA, United States) were used for multiplex immunohistochemistry (mIHC). Anti-mouse or anti-rabbit secondary antibodies contained TSA-488, TSA-555, Alexa-647 or Alexa-750 labels.

Multiplex immunohistochemistry was performed as described by Blom et al 2017 [2], with some modifications. Briefly, paraffin was removed from 3.5-µm FFPE sections and heat-induced epitope retrieval (HIER) was performed. After HIER, endogenous peroxide activity and protein blocking was performed in 0.9% H_2O_2 and in 10% normal goat serum, respectively. After the first primary antibody and a species-specific HRP-conjugated secondary antibody incubations, a tyramide signal amplification (TSA) (AlexaFluor488) (PerkinElmer, Waltham, MA) was done on the slides according to manufacturer´s instructions, and the reaction was incubated exactly 15 min. HIER was performed to denature the antibody complex and to attenuate the enzymatic activity of HRP. Another TSA reaction was done for the second primary-secondary antibody complex (AlexaFluor555). Then, after another HIER incubation, a pair of primary antibodies raised in different species was used to detect additional two targets using AlexaFluor647 and AlexaFluor750 fluorochrome-conjugated secondary antibodies. Nuclei were counterstained using DAPI and slides were mounted and coverslips applied.

Digital, whole-slide fluorescence images of mIHC slides were acquired at 0.32 µm/pixel resolution using Metafer 5 system including Axio Imager.Z2 microscope (Zeiss, Germany) equipped with EC Plan-Neofluar 20x objective (NA 0.8), Metafer scanning platform with CoolCube 2 CCD camera (MetaSystems, Germany), PhotoFluor LM-75 metal halide light source (89 North, Williston

VT), and DAPI, FITC, CY3, CY5, and CY7 filter sets. After image acquisition, images were converted to 8-bit JPEG2000 format (95% quality).

All image analyses were performed using CellProfiler (version 2.2.0). Cell classes were determined using pixel co-localization analysis. First, each channel intensity was thresholded using Adaptive Otsu, which is an automatic threshold selection method built in the CellProfiler software. Briefly, the Otsu algorithm returns a single intensity threshold that separates pixels into two classes, foreground and background. Double or triple channel positive pixels were determined with "MaskImage". Then, thresholded channel pixel areas were determined with "MeasureImageAreaOccupied" and areal proportions were counted by dividing the area with pixel area occupied by all the channels combined (ImageMath Add command). Cell class areas were exported as CSV files with "ExportToSpreadsheet". The quality of TMA cores was determined by visual inspection. TMA cores with low quality (e.g. ruptured or folded tissue or staining artefact) were excluded from further analyses.

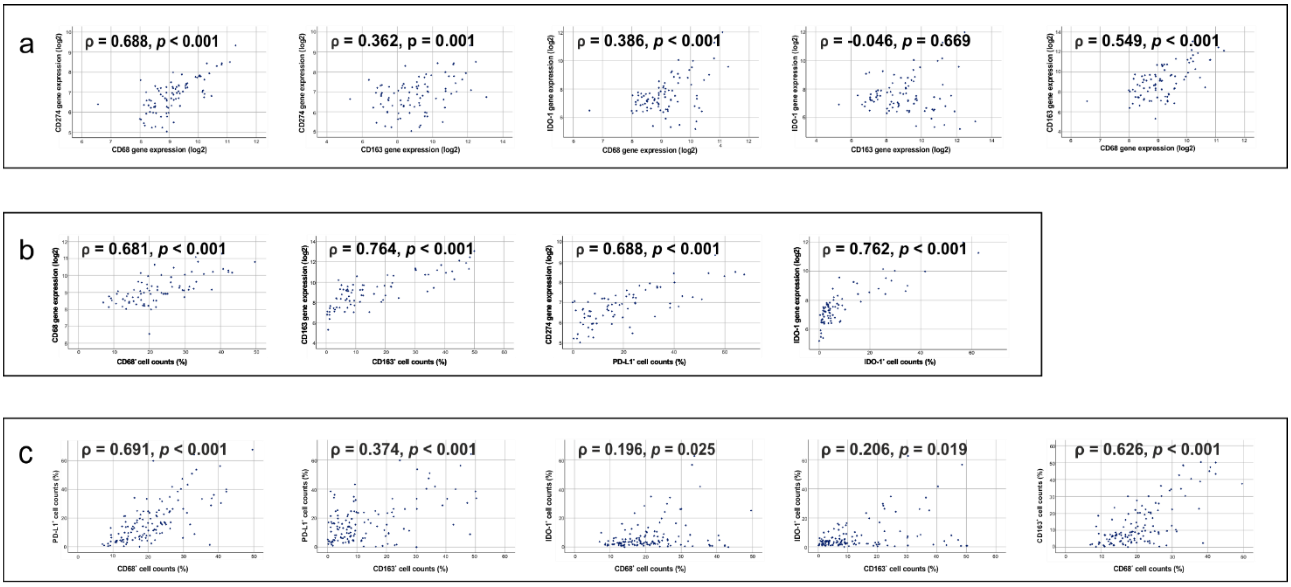

Figure S1. Correlation by Spearman rank analysis between **(a)** gene expression levels of macrophage markers, *CD274* and *IDO-1* **(b)** gene and protein expression levels of macrophage markers, *CD274*/PD-L1 and IDO-1 and **(c)** protein expression levels of macrophage markers, PD-L1 and IDO-1.

Table S1. Correlations by spearman rank analysis between interferon gamma gene expression levels and different cell proportions in the mIHC analysis.

***IFNG* vs cell immunophenotype**	**Spearman rho**	**p-val**
PD-L1$^+$	0.575	<0.001
IDO-1$^+$	0.687	<0.001
PD-L1$^+$CD68$^+$	0.613	<0.001
PD-L1$^+$CD163$^+$	0.630	<0.001
IDO-1$^+$CD68$^+$	0.643	<0.001
IDO-1$^+$CD163$^+$	0.678	<0.001

References

1. Kolstad, A.; Nome, O.; Delabie, J.; Lauritzsen, G.F.; Fossa, A.; Holte, H. Standard CHOP-21 as first line therapy for elderly patients with Hodgkin's lymphoma. *Leuk Lymphoma* **2007**, *48*, 570–576, doi:10.1080/10428190601126610.
2. Blom, S.; Paavolainen, L.; Bychkov, D.; Turkki, R.; Maki-Teeri, P.; Hemmes, A.; Valimaki, K.; Lundin, J.; Kallioniemi, O.; Pellinen, T. Systems pathology by multiplexed immunohistochemistry and whole-slide digital image analysis. *Sci. Rep.* **2017**, *7*, 15580, doi:10.1038/s41598-017-15798-4.

© 2020 by the authors. Licensee MDPI, Basel, Switzerland. This article is an open access article distributed under the terms and conditions of the Creative Commons Attribution (CC BY) license (http://creativecommons.org/licenses/by/4.0/).
